# Prediction of risk stratification in acute pulmonary embolism using a combined CTPA radiomics and machine learning model

**DOI:** 10.1097/MD.0000000000049227

**Published:** 2026-06-12

**Authors:** Jianxia Song, Yaxi Yu, Min Wang, Rong Chen, Yang Lu, Dawei Wang, Fei Wang, Fei Yang

**Affiliations:** a Graduate School of Hebei North University, Zhangjiakou, Hebei, China; b Department of Medical Imaging, the First Affiliated Hospital of Hebei North University, Zhangjiakou, Hebei, China; c Department of Thoracic Surgery, the First Affiliated Hospital of Hebei North University, Zhangjiakou, Hebei, China.

**Keywords:** acute pulmonary embolism, computed tomography pulmonary angiography, machine learning, radiomics features, risk stratification

## Abstract

This study extracted radiomics features of blood clots from computed tomography pulmonary angiography (CTPA) images of acute pulmonary embolism (APE) patients, and constructs a predictive model for APE patient risk stratification by comparing multiple machine learning (ML) algorithms. This retrospective study analyzed patients with APE, documenting their clinical characteristics (clinical and hematological indicators), conventional CTPA imaging parameters. Patients are stratified into low-risk group and high-risk group based on risk stratification. The data were randomly divided into a training cohort and a validation cohort in a 7:3 ratio. We developed 2 distinct models: nomogram model based on clinical characteristics and an image parameter model based on conventional CTPA imaging parameters. Features were screened by the least absolute shrinkage and selection operator (LASSO) method. The radiomics predictive model was constructed using 7 ML algorithms and selected the one with the best performance.The combined model was constructed by integrating radiomics features with clinical characteristics and image parameter using an optimal algorithm. Model performance was evaluated using receiver operating characteristic curves and decision curves analysis, with the area under the curve (AUC) compared via the DeLong test. A total of 202 patients were included. The training cohort contained 141 cases, while the validation cohort included 61 cases. Multivariate logistic analysis revealed that the pulmonary embolism severity index, dyspnea, troponin, and right ventricle/ left ventricle short-axis maximum diameter ratio were independent clinical predictors (*P* < .05). A total of 12 radiomics features were selected through LASSO screening. Logistic regression demonstrated the best performance among the 7 ML algorithms. In the validation cohort, the combined model demonstrated significantly superior predictive performance (AUC = 0.935) compared to the nomogram model (AUC = 0.805), the image parameter model (AUC = 0.788), and the radiomics model (AUC = 0.860) (*P* < .05). Decision curve analysis indicated that the combined model demonstrated higher clinical net benefit when the risk threshold exceeded 0.04. Calibration curves and Hosmer-Lemeshow test further confirmed the combined model’s goodness of fit in both the training and validation cohorts. The combined model combining clinical characteristics, conventional CTPA imaging parameters, and radiomics features demonstrated outstanding predictive performance and clinical applicability in risk stratification for APE patients.

## 1. Introduction

Acute pulmonary embolism (APE), a major cardiovascular emergency with high incidence and mortality rates, poses a persistent challenge to global public health.^[1,2]^ The clinical course is significantly heterogeneous, ranging from asymptomatic to life-threatening circulatory failure. Therefore, early and precise risk stratification is a core component of optimizing clinical management, guiding treatment decisions (such as thrombolysis or anticoagulation), and improving patient outcomes.^[3]^ Computed tomography of the pulmonary artery (CTPA) is currently the first-line imaging modality for patients with suspected APE owing to its high sensitivity and specificity. In addition to directly visualizing thrombi, CTPA can also provide quantitative parameters such as the right ventricular short-axis maximum diameter/left ventricle short-axis maximum diameter (RV/LV) ratio. Currently, international guidelines primarily rely on CTPA-based indicators of right ventricular dysfunction (RV/LV ≥ 1.0), clinical scoring systems (such as pulmonary embolism severity index, PESI), and serum biomarkers (such as troponin) for risk stratification.^[4–7]^ However, while these conventional indicators possess some predictive value, their limited information dimensions make it difficult to comprehensively capture the biological heterogeneity of thrombi and the complex pathophysiological connections between thrombi and clinical outcomes. With advances in medical imaging analysis technology, radiomics has emerged over time. It can extract and quantify the heterogeneity of morphology, texture, and spatial distribution that cannot be recognized by the human eye through high-throughput imaging,^[8]^ and thus noninvasively characterize the internal heterogeneity of a thrombus. Machine learning (ML), a vital branch of artificial intelligence (AI), can identify complex patterns among high-dimensional features and construct robust predictive models.^[9]^ The Pulmonary Embolism Research Collaborative proposed that ML plays an increasingly important role in facilitating the diagnosis of APE.^[10]^ However, existing studies generally lack systematic comparisons and validations of multiple ML algorithms. Moreover, the effective integration of radiomics with ML algorithms to construct a high-performance, clinically practical, and combined predictive model remains a key issue that has not been fully addressed in current research. Therefore, this study aimed to develop a comprehensive predictive model integrating clinical characteristics, conventional CTPA imaging parameters, and thrombus radiomics features using the optimal ML algorithm to achieve more precise risk stratification in patients with APE.

## 2. Materials and methods

### 2.1. Study population

Retrospectively collected patients who underwent CTPA examinations and were diagnosed with APE at the First Affiliated Hospital of North Hebei University from January 2021 to September 2025.

Inclusion criteria: Meet relevant diagnostic criteria for APE and confirmed APE by CTPA (imaging showing low-density filling defects in the pulmonary artery lumen or narrowing and occlusion of the pulmonary artery lumen).^[11]^

The exclusion criteria were as follows: a history of chronic pulmonary embolism or embolism in other organs; severe cardiac disease, including valvular disease, cardiomyopathy, or congenital heart disease; severe hepatic or renal insufficiency; incomplete clinical data or missing follow-up information; presence of malignant tumors; and incomplete CTPA images or severe artifacts.

Grouped: Patients were categorized into high-risk, intermediate-high-risk, intermediate-low-risk, and low-risk groups based on the European Society of Cardiology expert consensus.^[3]^ Based on clinical treatment decisions, patients in the intermediate-low-risk and low-risk groups are typically treated with anticoagulation therapy alone, with a relatively short treatment duration, whereas patients in the high-risk and intermediate-high-risk groups should typically receive a comprehensive treatment regimen combining reperfusion therapy with anticoagulation therapy, accompanied by enhanced in-hospital monitoring and long-term follow-up. Therefore, this study classified the low-risk and intermediate-low-risk groups as the low-risk group and the intermediate-high-risk and high-risk groups as the high-risk group.

**Figure 1.**
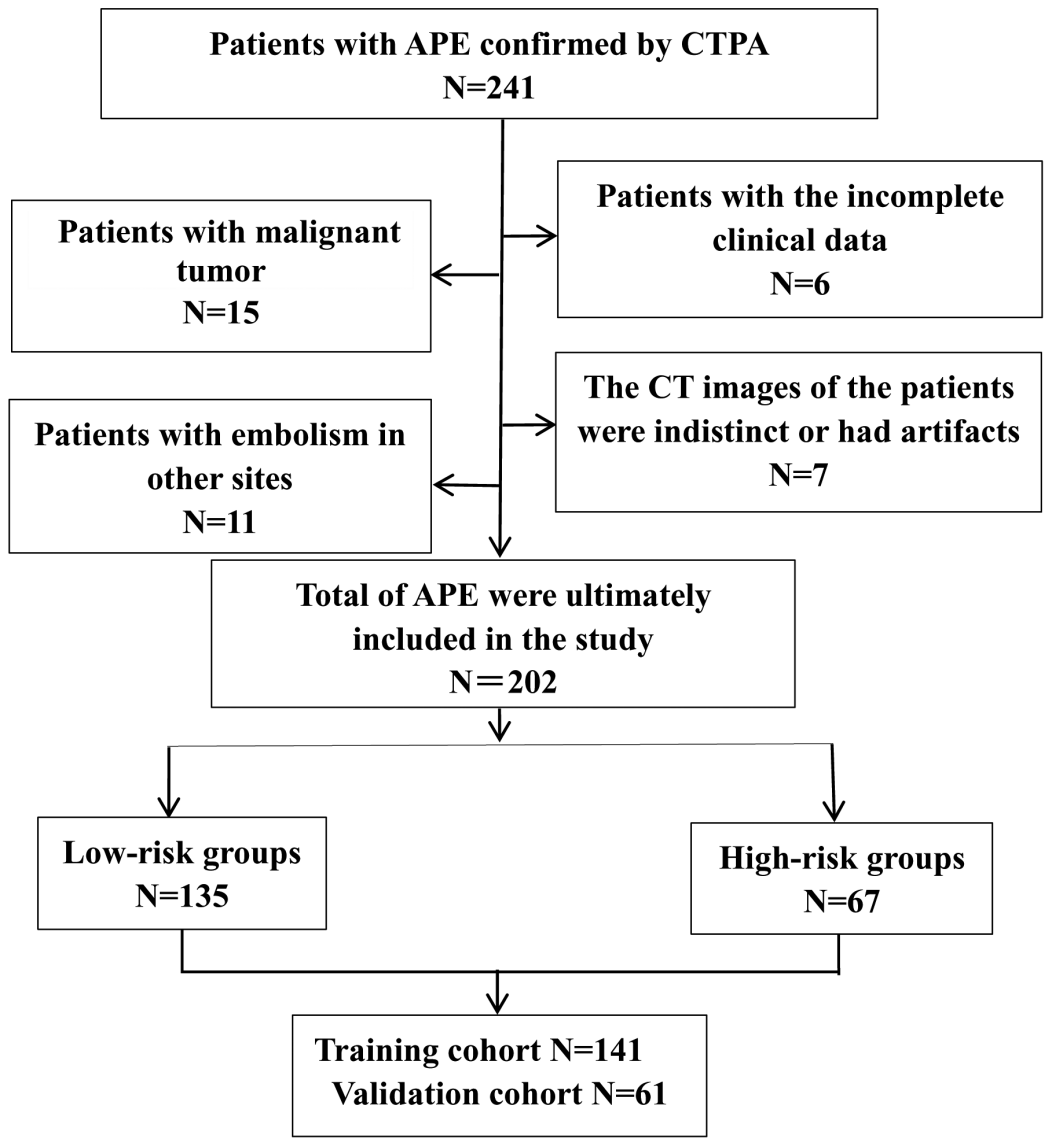
Flow sheet of patient selection.

This study enrolled 202 patients, 135 in the low-risk group and 67 in the high-risk group (Fig. 1). A total of 202 patients were randomly assigned in a 7:3 ratio to the training cohort (141 patients) and validation cohort (61 patients).

### 2.2. CTPA acquisition

All the patients were scanned using a Japanese 320-slice CT scanner. The scan parameters were as follows: tube voltage was set to 100 kVp and manually adjusted to 120 kVp upon reaching the maximum automatic current, with the tube current controlled by the automatic exposure control system. The threshold for the pulmonary artery trunk was set at 200 HU; once the region of interest exceeded this threshold, images were automatically captured after a 3-second delay. Contrast agent: iodixanol (320 mgI/mL, dose 0.8 mL/kg, rate 4 mL/s), followed by a 30 mL saline flush.

### 2.3. Clinical indicators collection and hematological indicators detection

The patients’ clinical indicators included age, sex, smoking history, hypertension, diabetes, dyspnea, hemoptysis, pectoralgia, and syncope. Peripheral venous blood was collected from the patients for laboratory testing. The samples were collected in test tubes containing ethylenediaminetetraacetic acid. Five milliliters of blood were used for complete blood count analysis, including white blood cell, lymphocyte, neutrophil, and hemoglobin counts. Additionally, 3 mL of blood was used to measure the plasma D-dimer levels using a fully automated coagulation analyzer (CS5100). Subsequently, PESI scores were calculated.

### 2.4. Measurements of RV/LV ratio (Fig. 2)

**Figure 2.**
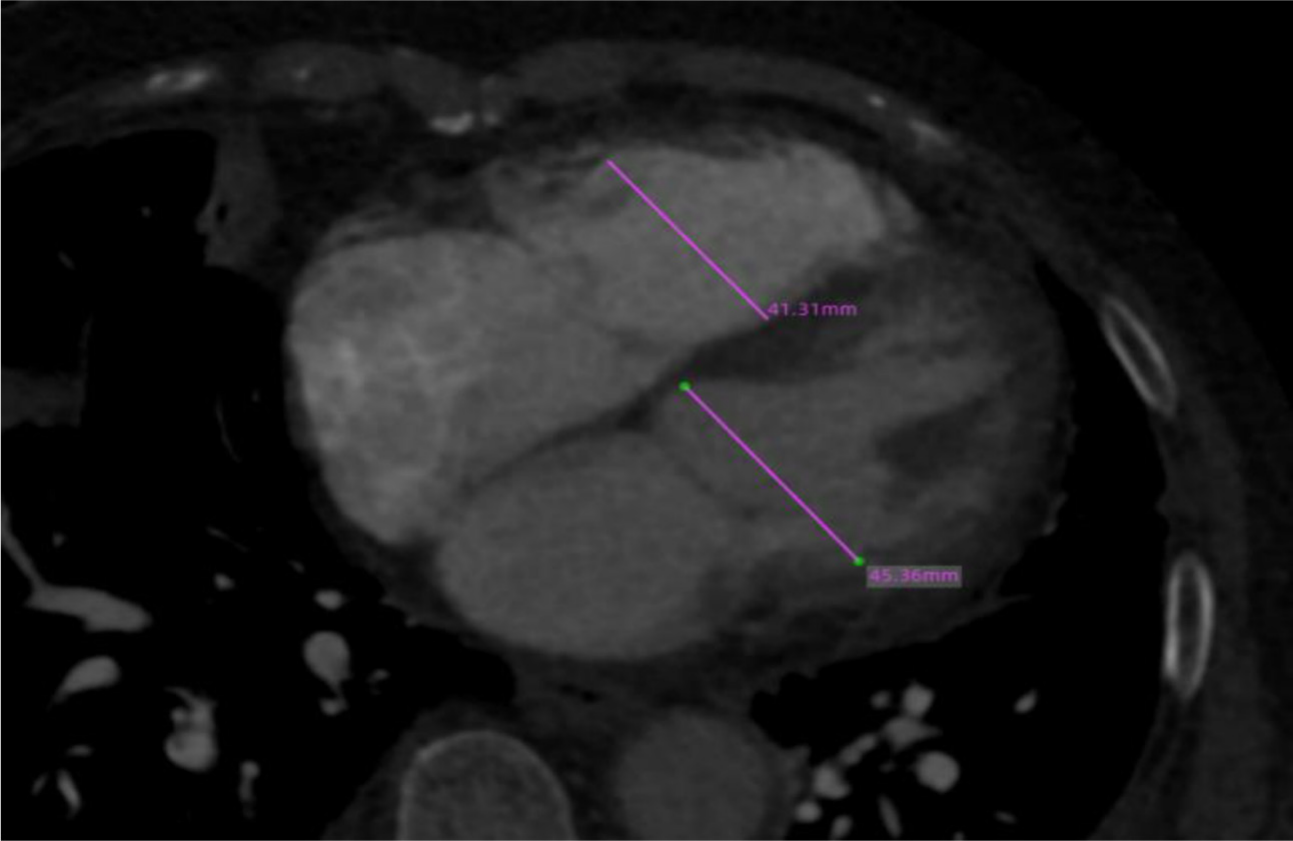
Measurement of RV and LV.

The window width and level were adjusted to appropriate settings for the CTPA axial images. The maximum short-axis diameters of the right and left ventricles were measured and their ratios were calculated (Fig. 2). For the right ventricular short-axis maximum diameter, the distance from the inner wall of the right ventricular free wall to the inner wall of the interventricular septum was at the widest point at the tricuspid valve level. For the left ventricular short-axis maximum diameter, the distance from the inner wall of the left ventricular free wall to the inner wall of the interventricular septum was at the widest point at the mitral valve level.^[12]^

### 2.5. Image processing and segmentation

**Figure 3.**
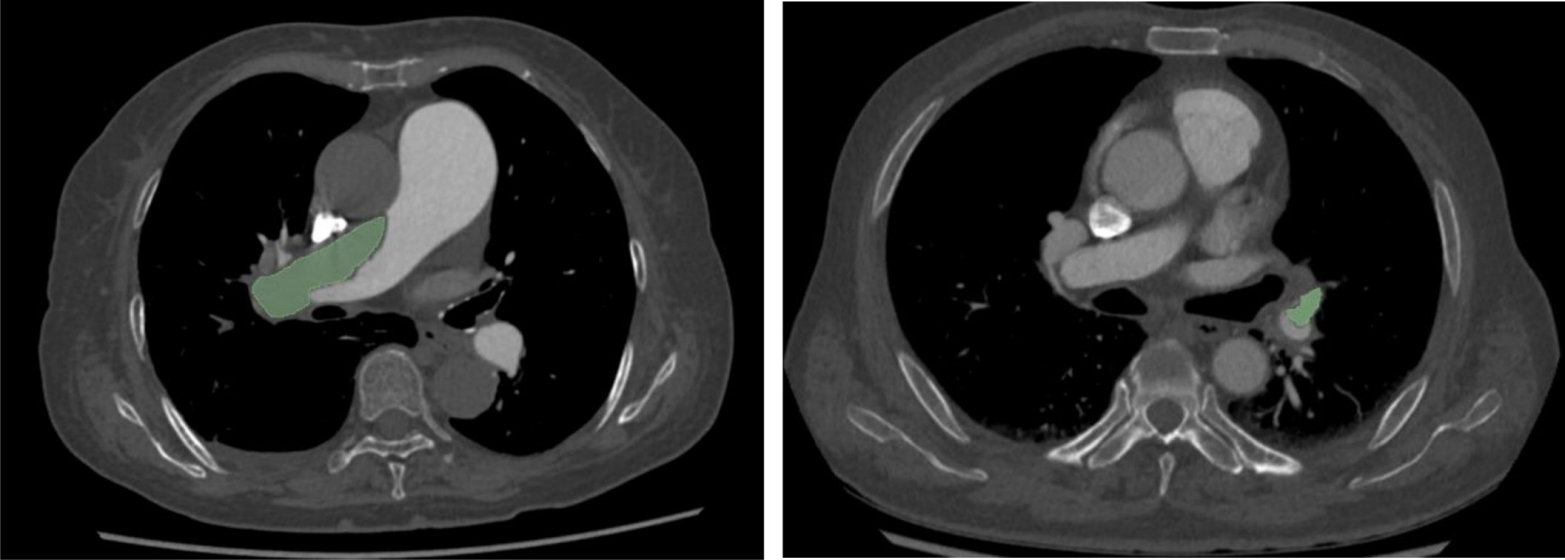
The ROI is outlined along the edge of the lesion.

The patients’ CTPA images were imported into the 3D-Slicer software in the DICOM format. Regions of interest (ROI) was manually delineated along the thrombus margins on each patient’s CTPA arterial-phase image. The ROI was ensured to avoid areas of artifacts within the embolus and to maintain a 2 mm margin from the surrounding normal tissue (Fig. 3). All CTPA image segmentations for patients were performed independently by 2 radiologists with more than 5 years of experience in chest imaging diagnosis, using a blinded approach. To assess segmentation consistency, all participants underwent systematic training on imaging ROI delineation consensus before formal segmentation and completed practice delineation exercises in pre-experimental cases. This ensured a uniform understanding of the segmentation standards. All radiologists declared no conflicts of interest. CT images from 20% of patients were randomly selected for independent delineation by 2 physicians. The consistency and repeatability of feature extraction between the observers were quantified using the intraclass correlation coefficient (ICC).

### 2.6. Extraction, screening, and model construction of radiomics features

After contouring was completed, image feature parameters were extracted, including those based on the Gray-Level Co-occurrence Matrix (GLCM), Gray-Level Size Zone Matrix (GLSZM), Gray-Level Run Length Matrix (GLRLM), Gray-Level Dependence Matrix (GLDM), Neighboring Gray-Tone Difference Matrix, as well as shape and first-order features.

Radiomics features were standardized, and the least absolute shrinkage and selection operator (LASSO) was applied to select the most predictive features within the training cohort. Logistic regression (LR), Random Forest (RF), k-nearest neighbor (KNN), Extreme Gradient Boosting (XGBoost), Light Gradient Boosting Machine (LightGBM), Naïve Bayes (NB), and Decision Tree (DT) were employed to establish the radiomics prediction models, retaining the model with the best performance.

### 2.7. Constructing of the nomogram and image parameter models

Potential predictors of APE risk stratification were identified using univariate LR analysis of the patients’ clinical characteristics and conventional imaging parameters. The characteristics of univariate analysis (*P *< .05) were included in multivariate LR analysis to identify independent predictors and establish clinical nomogram and imaging parameter models.

### 2.8. Construction of the combined prediction model

We integrated independent clinical and imaging predictors with radiomics features to build a combined prediction model using the best ML algorithm, which was then validated in the validation cohort.

### 2.9. Statistical methods

Statistical analyses of clinical characteristics and imaging parameters were performed using SPSS 27.0 and R4.1.1. Normality of the measurement data was assessed using the Shapiro–Wilk test. Normally distributed data were presented as mean ± standard deviation, while non-normally distributed data were expressed as medians with interquartile ranges. Differences between groups were assessed using either an independent samples t-test or a nonparametric test for continuous variables. Categorical data were expressed as proportions, and group comparisons were made using the chi-square or Fisher exact test. Statistically significant indicators (*P* < .05) were incorporated into the multivariate LR model. Receiver operating characteristic (ROC) curves were plotted for each model, and the area under the curve (AUC), optimal cutoff value, sensitivity, and specificity were calculated. The DeLong test was used to compare the statistical differences in AUC values among the predictive models. Calibration curves were plotted to assess the model’s goodness of fit. The Hosmer–Lemeshow test was used to evaluate the calibration curve reliability. Decision curve analysis (DCA) quantified the clinical utility across the 4 models by calculating the net benefit. Statistical significance was set at *P* < .05.

## 3. Results

### 3.1. Analysis of patients’ clinical characteristics and imaging parameters

Table 1 presents the clinical indicators of the patients in the training cohort. The hematological indicators and conventional imaging parameters are presented in Tables 2 and 3. Variables including smoking status, dyspnea, PESI, white blood cell count, neutrophil count, neutrophil-to-lymphocyte ratio, hemoglobin, troponin, and the conventional imaging parameter RV/LV differed significantly between the low- and high-risk groups (*P* < .05).

**Table 1:** Comparison of clinical indicators among patients in the training cohort (n = 141).

Clinical indicators		Low-risk group (n = 96)	High-risk group (n = 45)	Z/X^2^	*P*
Gender	Male	49 (51.0)	22 (48.9)	0.057	.812
	Female	47 (49.0)	23 (51.1)		
Smoking	Yes	23 (24.0)	23 (51.1)	7.360	<.05
	No	73 (76.0)	24 (48.9)		
Age		66 (13.0)	70 (10.5)	−2.948	.914
Hypertension	Yes	38 (39.6)	21 (46.6)	0.632	.427
	No	58 (60.4)	38 (84.4)		
Diabetes	Yes	13 (13.5)	5 (11.1)	0.163	.687
	No	83 (86.5)	40 (88.9)		
Dyspnea	Yes	18 (18.7)	17 (37.8)	5.944	<.05
	No	78 (81.3)	28 (62.2)		
Hemoptysis	Yes	11 (11.5)	4 (8.9)	0.213	.645
	No	85 (88.5)	41 (91.1)		
Pectoralgia	Yes	19 (19.8)	11 (24.4)	0.396	.529
	No	77 (80.2)	34 (75.6)		
Syncope	Yes	11 (11.5)	10 (22.2)	2.801	.094
	No	85 (88.5)	35 (77.8)		
Pulse		83 (23.5)	92 (26.5)	−1.305	.192
Troponin	Normala	83 (86.5)	28 (62.2)	10.744	<.05
	Bnormal	13 (13.5)	17 (37.8)		

**Table 2:** Hematological indicators of patients in the training cohort (n = 141).

Hematological indicators	Low-risk group (n = 96)	High-risk group (n = 45)	Z/F	*P*
White blood cell count	6.36 (2.50)	8.34 (4.13)	−4.332	<.001
Neutrophil count	4.12 (2.23)	6.17 (3.94)	−4.806	<.001
Lymphocyte count	1.49 (0.83)	1.51 (0.87)	−0.380	.704
Monocyte count	0.41 (0.18)	0.39 (0.34)	−0.062	.951
Hemoglobin	139.00 (23.00)	147.00 (34.00)	−2.163	<.05
D-dimer	5.31 (5.46)	5.80 (6.14)	−1.272	.204
NLR	2.63 (2.25)	3.99 (5.22)	−3.702	<.001
PESI	79.08 ± 19.05	94.51 ± 14.83	1.057	<.05

**Table 3:** Analysis of imaging parameters in the training cohort (n = 141).

Parameters	Low-risk groups (n = 96)	High-risk groups (n = 45)	X^2^	*P*
RV/LV			6.361	<.05
≥1.0	40 (41.7)	29 (64.4)		
<1.0	56 (58.3)	16 (35.6)		

### 3.2. Univariate and multivariate LR analysis (Tables 4–5)

Univariate and multivariate LR analyses revealed that dyspnea, troponin level, PESI, and RV/LV ratio were independent predictors of risk stratification in patients with APE (Tables 4 and 5). Collinearity diagnostics showed that the tolerance values for all clinical independent variables exceeded 0.1, while the variance inflation factors remained below 5, indicating no significant multicollinearity among the predictive variables.

**Table 4:** Univariate regression analysis.

Parameter	B	OR	95%CI	*P*
PESI	0.048	1.049	1.025–1.072	<.001
White blood cell count	0.313	1.367	1.170–1.598	<.001
Neutrophil count	0.347	1.415	1.202–1.665	<.001
NLR	0.167	1.182	1.062–1.315	<.05
Hemoglobin	0.022	1.022	1.005–1.040	<.05
Smoking	1.021	2.777	1.312–5.880	<.05
Dyspnea	0.967	2.631	1.193–5.803	<.05
Troponin	1.355	3.876	1.674–8.976	<.001

**Table 5:** Multivariate regression analysis.

Parameter	B	OR	95%CI	*P*
Smoking	0.428	1.534	0.574–4.099	.393
Troponin	1.213	3.363	1.126–10.039	<.05
Dyspnea	1.025	2.786	1.050–7.394	<.05
PESI	0.046	1.047	1.019–1.076	<.001
White blood cell count	-0.255	0.775	0.294–2.042	.606
RV/LV	0.931	2.537	1.219–5.281	<.05

### 3.3. Construction of nomogram model (Fig. 4) and conventional image parameter model

**Figure 4.**
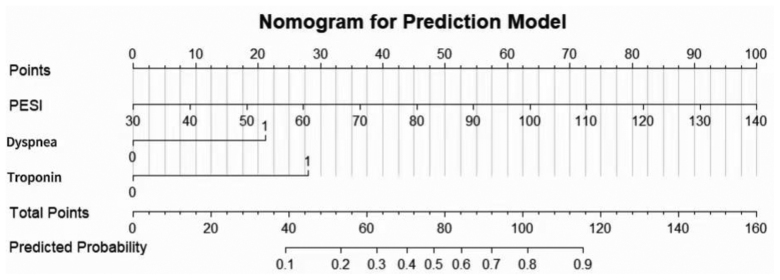
Nomogram model.

A nomogram model incorporating dyspnea, troponin, and PESI was constructed to predict the risk stratification in patients with APE (Fig. 4). A conventional imaging parameter model based on the RV/LV ratio was developed to predict the risk stratification in patients with APE. ROC curve analysis revealed that the AUC of the nomogram model in the training cohort was 0.834 (95% confidence interval [CI]: 0.766–0.903), sensitivity was 91.1%, and specificity was 74.0%; the AUC of the model in the validation cohort was 0.805 (95% CI: 0.690–0.920), sensitivity was 86.4%, and specificity was 66.7%.

### 3.4. Radiomics features extraction

**Figure 5.**
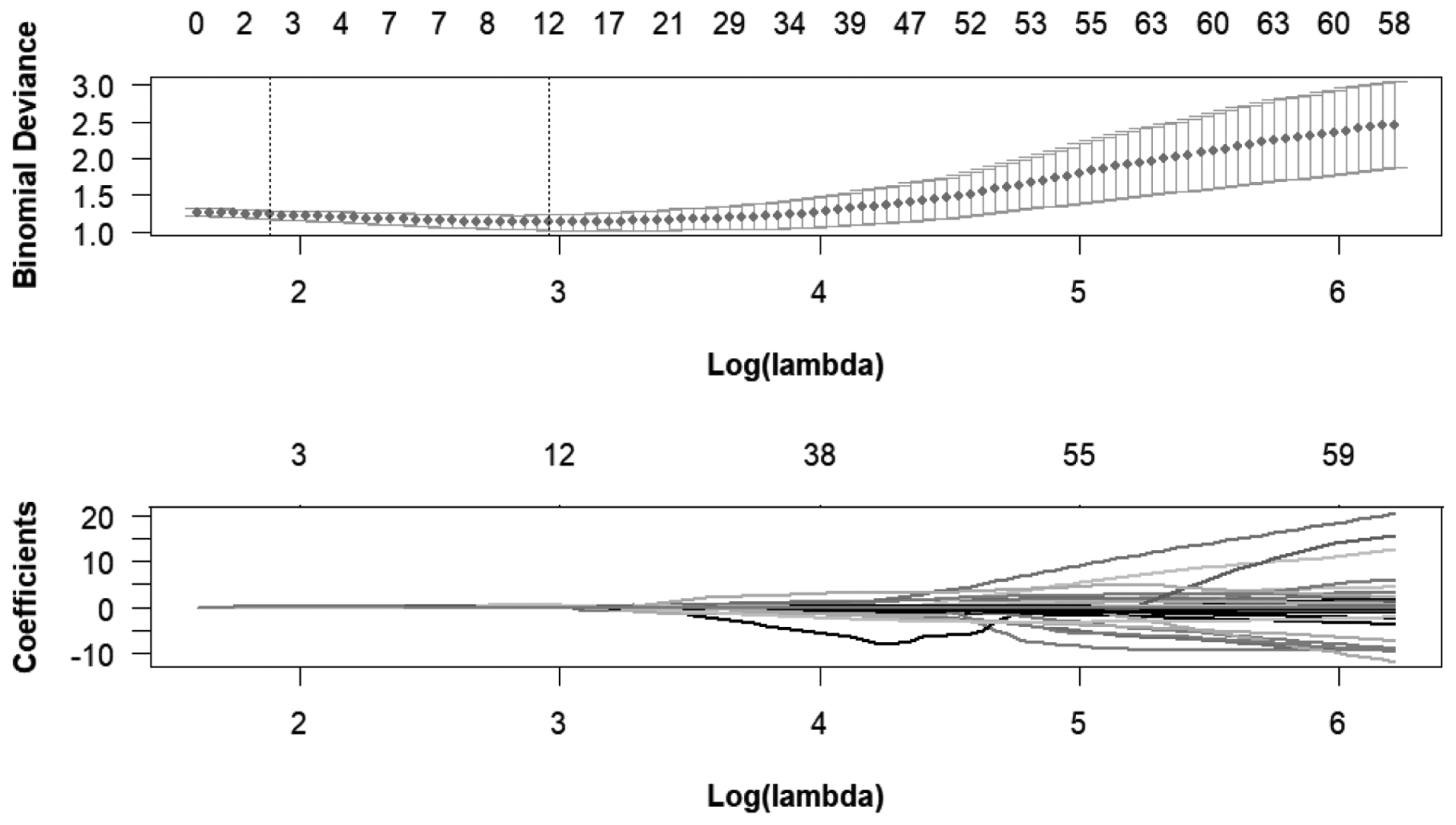
Feature reduction and screening process using LASSO regression and tenfold cross-validation.

A total of 944 radiomics features were extracted. Using LASSO regression and 10-fold cross-validation, 12 non-zero coefficient features were selected when λ = 0.052 (Fig. 5): major-axis-length, median, maximum.2, Run-Entropy.2, Large-Area-Low-Gray-Level-Emphasis.2, Skewness.4, Gray-Level-Non-Uniformity.12, Autocorrelation.5, Inverse-Variance.5, 10Percentile.7, Skewness.7, Large-Dependence-High-Gray-Level-Emphasis.7. Additionally, ICC analysis revealed an average interobserver correlation coefficient of 0.978 (95% CI: 0.958–0.988, *P* < .001), indicating excellent interobserver agreement and ensuring the reliability of feature extraction.

### 3.5. Construction of Radiomics model

The specific results of the 7 ML-radiomics models (LR, RF, KNN, XGBoost, LightGBM, NB, and DT) are listed in Table 6. The radiomics model based on LR demonstrated the best performance with AUC values of 0.872 and 0.860 for the training and validation cohorts, respectively.

**Table 6:** ROC curve analysis of 7 ML-radiomics models.

	AUC	95% CI	Sensitivity	Specificity	Accuracy	Cutoff value/Optimal Iterations	PPV	NPV
**Training cohort**								
LR	0.872	0.809–0.922	1.000	0.583	0.716	0.118	0.529	1.000
RF	0.749	0.622–0.833	0.689	0.719	0.709	0.426	0.534	0.831
KNN	0.846	0.831–0.862	0.713	0.812	0.755	0.589	0.835	0.680
XGBoost	0.853	0.834–0.871	0.718	0.831	0.769	0.609	0.836	0.711
LightGBM	0.500	0.500–0.500	0.505	0.506	0.505	39	0.550	0.460
DT	0.776	0.695–0.852	0.622	0.885	0.801	0.283	0.718	0.833
NB	0.828	0.756–0.900	0.487	0.927	0.787	0.572	0.759	0.795
**Validation cohort**								
LR	0.860	0.752–0.950	0.864	0.744	0.787	0.292	0.655	0.906
RF	0.751	0.599–0.892	0.727	0.872	0.820	0.480	0.762	0.850
KNN	0.860	0.845–0.874	0.768	0.767	0.768	0.840	0.813	0.715
XGBoost	0.853	0.834–0.872	0.808	0.750	0.784	0.566	0.826	0.727
LightGBM	0.500	0.500–0.500	0.504	0.506	0.505	29	0.598	0.413
DT	0.832	0.724–0.925	0.727	0.923	0.852	0.521	0.842	0.857
NB	0.826	0.707–0.945	0.818	0.641	0.705	0.599	0.562	0.862

LR = logistic regression, NPV = negative predictive value, PPV = positive predictive value.

### 3.6. Predictive performance and clinical utility of combined model for risk stratification in patients with APE

**Figure 6.**
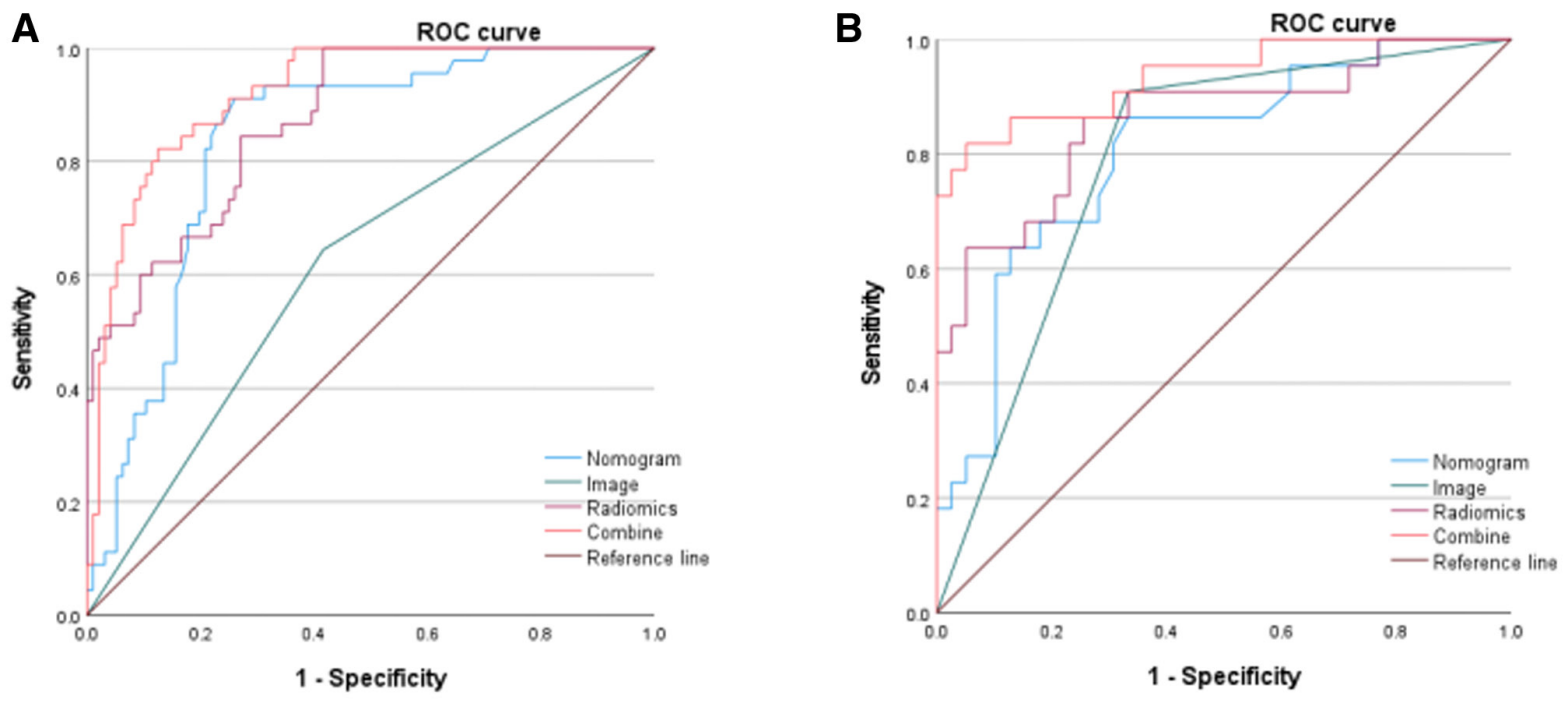
ROC curves of the ML-radiomics model, nomogram model, image parameter model, and combined model in the training cohort (A) and validation cohort (B).

**Figure 7.**
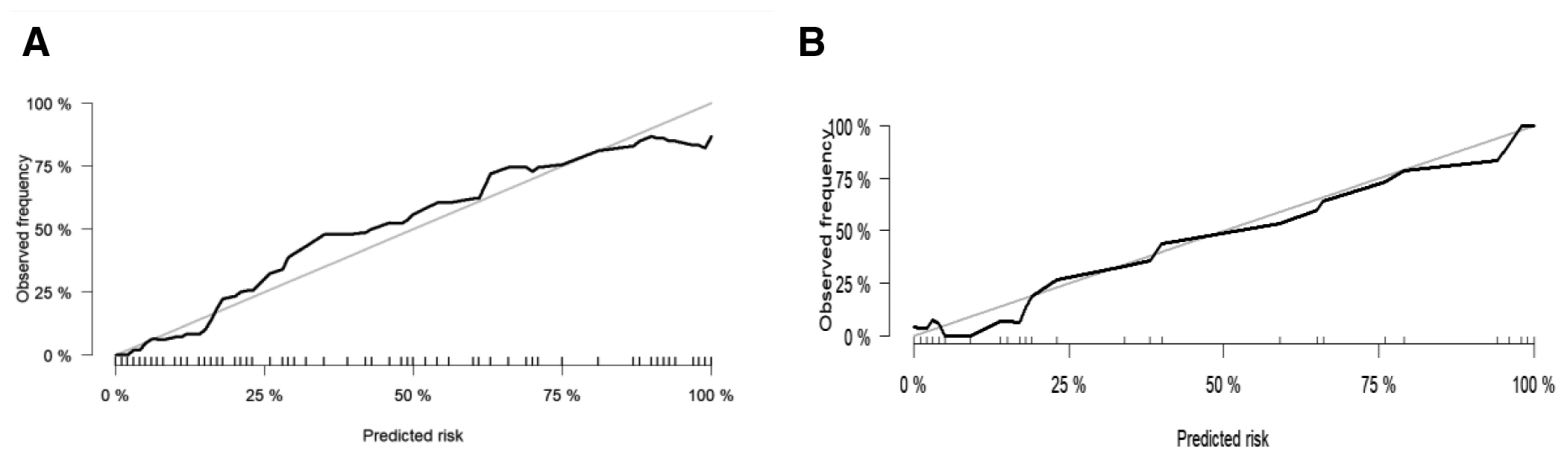
Calibration curves of the combined model in the training cohort (A) and validation cohort (B).

**Figure 8.**
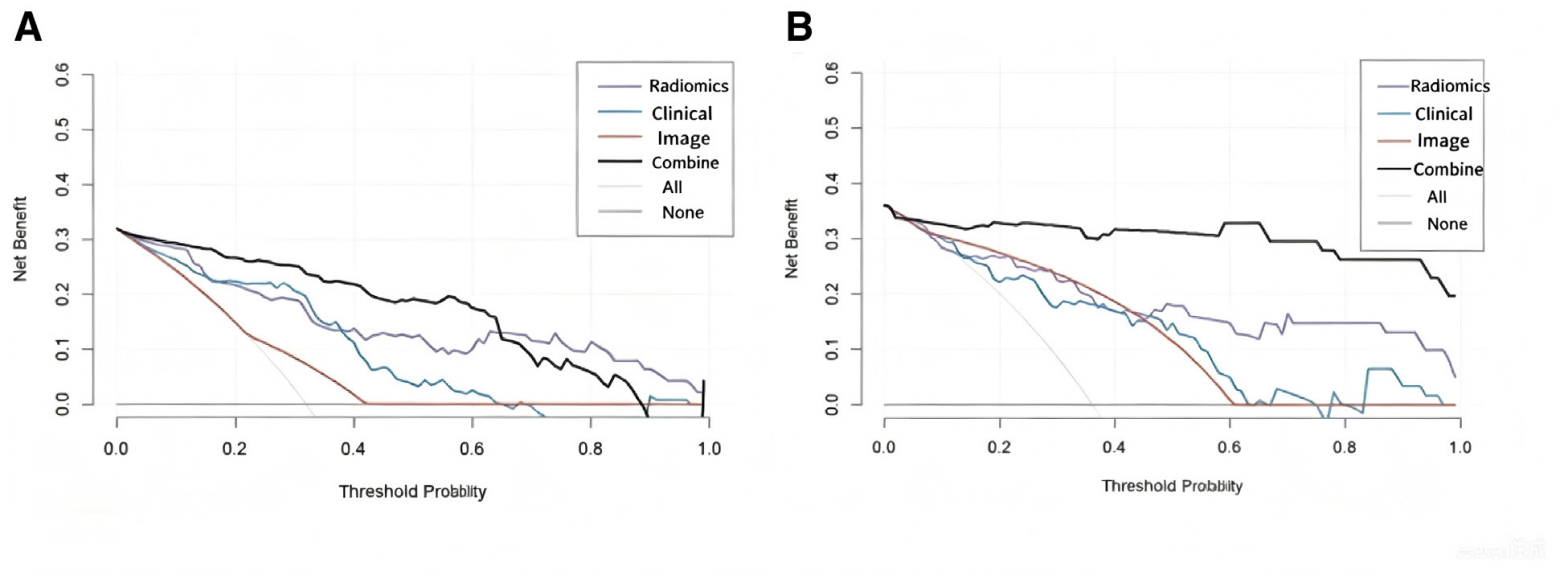
Decision curve analysis of the combined prediction model for training cohort (A) and validation cohort (B).

In the training cohort, a combined model incorporating radiomics features, 3 clinically independent predictors (dyspnea, troponin, and PESI), and conventional imaging parameter (RV/LV) was established using LR. The AUC for the training and validation cohorts were 0.920 and 0.935, respectively (Table 7 and Fig. 6). According to the DeLong test, in the training cohort, the combined model outperformed the nomogram model, ML-radiomics model, and imaging parameter model (*Z* = −2.639, *P* = .008; *Z* = −2.390, *P* = .017; *Z* = −6.656, *P* = .000). In the validation cohort, the combined model also outperformed the nomogram model, ML-radiomics model, and imaging parameter model (*Z* = −2.838, *P* = .005; *Z* = −1.992, *P* = .046; *Z* = −3.322, *P* = .001) (Table 8). In both the training and validation cohorts, the calibration curves graphically demonstrated good agreement between the predicted and actual outcomes of the combined model (Fig. 7). The Hosmer-Lemeshow test further confirmed the satisfactory goodness-of-fit of the combined model in both cohorts (*P* = .114 for training, *P* = .354 for validation). The DCA indicated that, in the validation cohort, the optimal threshold for the combined model was 0.04; therefore, for the combined model, we selected a risk probability > 0.04 as the final application criterion (Fig. 8).

**Table 7:** ROC curve analysis of severity in APE patients.

	Model	AUC	95%CI	Sensitivity	Specificity	Cut off value	*P*
Training cohort	Nomogram	0.834	0.766–0.903	0.911	0.740	0.291	<.05
	Image	0.614	0.515–0.713	0.644	0.583	0.500	<.05
	ML-radiomics	0.872	0.809–0.922	1.000	0.583	0.304	<.05
	Combine	0.920	0.876–0.964	0.822	0.875	0.391	<.001
Validation cohort	Nomogram	0.805	0.691–0.919	0.864	0.667	0.247	<.05
	Image	0.788	0.671–0.905	0.909	0.667	0.500	<.001
	ML-radiomics	0.860	0.752–0.950	0.864	0.744	0.292	<.05
	Combine	0.935	0.868–1.000	0.818	0.949	0.515	<.001

**Table 8:** DeLong test of 4 models.

		Z	95% CI	*P*	AUC diversity
Training cohort	Nomogram-Combine	−2.639	−0.149–−0.022	.008	−0.085
	Image-Combine	−6.656	−0.396–−0.216	.000	−0.306
	Radiomics-Combine	−2.390	−0.087–−0.009	.017	−0.048
Validation cohort	Nomogram-Combine	−2.838	−0.219–−0.040	.005	−0.129
	Image-Combine	−3.322	−0.233–−0.060	.001	−0.147
	Radiomics-Combine	−1.992	−0.148–−0.001	.046	−0.075

## 4. Discussion

This study successfully developed and validated an ML ensemble model that integrated clinical characteristics, conventional CTPA imaging parameters, and thrombus radiomics features for risk stratification in patients with APE. These findings confirm that multimodal information fusion generates synergistic effects, yielding a predictive tool significantly superior to any single information source. The combined model demonstrated an exceptional discriminatory ability in the independent validation cohort (AUC = 0.935). Its predictive performance significantly outperformed both the clinical characteristic-based nomogram model (AUC = 0.805) and the conventional imaging parameter model (AUC = 0.788), and surpassed the best ML-radiomics model relying solely on radiomics features (AUC = 0.860). This finding strongly indicates that while radiomics can uncover heterogeneous information within thrombi that is invisible to the naked eye, combining it with conventional indicators reflecting the patient’s overall physiological status (such as PESI scores and dyspnea) and right ventricular function (such as RV/LV ratios) is essential for constructing a more comprehensive and robust APE disease risk assessment system. This approach provides a more precise risk evaluation.

In recent years, the application of CTPA-based radiomics and ML techniques for APE risk stratification has attracted widespread attention. Gotta et al^[13]^ developed a ML classifier for APE risk stratification based on dual-energy CT (DECT) radiomics. In this study, over 1300 radiomics features were extracted from the DECT images. After screening, the resulting random forest model achieved an AUC of 0.82 in the validation cohort, demonstrating the incremental value of radiomics features in clinical risk scoring and stratification. Guo et al^[14]^ investigated the ability of the pulmonary artery obstruction index (PAOI) to assess risk stratification by comparing the differences in the PAOI among patients with APE in different risk groups. They ultimately found that the PAOI demonstrated good predictive performance for high-risk patients, suggesting that it is useful for risk stratification in patients with APE. Collomb et al^[15]^ conducted a retrospective analysis of APE severity in 81 patients. Multivariate analysis revealed that the PAOI, RV/LV ratio, central pulmonary artery diameter, and left ventricular short-axis diameter were significantly associated with the severity of APE. Previous studies have largely focused on single-dimensional radiological features, such as extracting only indicators related to thrombus burden and relying on manual calculations or traditional image analysis methods. These approaches have limitations, including a narrow scope of features, high subjectivity, and susceptibility to human error, which make it difficult to comprehensively reflect the severity of APE in patients. Additionally, they face issues such as insufficient model generalization and strong dependence on sample distribution. Wang et al^[16]^ compared the performance of various ML and deep learning (DL) algorithms based on CT radiomics features to predict short-term adverse outcomes in patients with APE. They found that both ML and DL models based on thrombus texture features demonstrated high predictive performance. However, that study focused on short-term adverse prognosis as its endpoint, whereas our study aimed to predict risk stratification. This study combined and compared radiomics with various ML algorithms for modeling, and constructed a combined model integrating radiomics features with key clinical characteristics. This approach not only addresses the issue of insufficient generalization ability in single algorithms but also overcomes the limitations of standalone radiomics or clinical models through multi-indicator fusion, thereby achieving optimization and innovation in modeling methods.

This study extracted 944 radiomics features from CTPA images and selected 12 features with the highest predictive value through LASSO regression. They encompass 3 dimensions: first-order statistics, textural features, and shape features. Specifically, they include 5 first-order features, 2 GLRLM features, 2 GLCM features, one GLSZM feature, one GLDM feature, and one shape feature. First-order statistical features reflect the distribution of CT values within the thrombus region, enabling the assessment of its composition, density, and homogeneity. Furthermore, parameters such as kurtosis and entropy reveal the internal structural heterogeneity of the thrombus, thereby providing a basis for evaluating APE severity. The Skewness.4 and Skewness.7 features revealed the internal heterogeneity of thrombi from a microscopic perspective of image signal stability. This heterogeneity suggests a higher potential risk of thrombus detachment and progression, which indirectly indicates a higher clinical risk. Textural features quantify the internal heterogeneity^[17]^ and spatial patterns of lesions and exhibit high homogeneity. The GLCM serves as a parameter for quantifying the correlation and complexity of the grayscale distribution in images.^[18,19]^ High-entropy values indicate complex irregular textures, potentially suggesting thrombus organization, recanalization, or mixed thrombus formation. Large-Area-Low-Gray-Level-Emphasis.2 (GLSZM) indicated that large gray-scale area matrices dominate, directly capturing the 2 key attributes of “large blocks” and “low density.” This automatically and objectively quantified the most hazardous components, signaling the acute nature of thrombosis and the instability of the condition. GLDM indicates the presence of large areas of uniform obstruction. Large-Dependence-High-Gray-Level-Emphasis.7 measured extensive uniform regions within the image, indicating large intact occlusive thrombi that are closely associated with acute high-risk conditions. Shape serves as a characteristic describing the morphology, size, and spatial distribution of the thrombus itself, enabling precise quantification of the thrombus burden and its distribution pattern. Major-axis-length belongs to the size and dimension categories of shape features and directly describes the physical spatial measurements of a region. This indicates the extent of an object’s extension along its primary axis and is crucial for identifying high-risk central embolisms. As a fundamental and vital morphological feature, it combines with first-order statistical features and texture features to collectively construct a comprehensive “portrait” of the thrombus. These features collectively constitute a quantitative description of the thrombus phenotype, which advances risk assessment from macroscopic anatomical localization to microscopic structural analysis.

In comparative studies of ML algorithms, we found that LR demonstrated an optimal and robust predictive performance, warranting further investigation. The radiomics model based on LR algorithm achieved AUC values of 0.872 (95% CI: 0.809–0.922) and 0.860 (95% CI: 0.782–0.876) in the training and validation cohorts, respectively, outperforming the other candidate models. This may be attributed to the fact that after rigorous feature selection, the limited number (12) of retained high-value features likely exhibited an approximately linear, separable relationship with clinical outcomes. In such scenarios, LR has advantages owing to its simplicity, resistance to overfitting, and high interpretability. Conversely, more complex ensemble algorithms, such as RF and XGBoost, may fail to fully capture complex nonlinear relationships with limited sample sizes and may even introduce unnecessary variance.

This study further validated the independent predictive value of indicators such as RV/LV ratio, troponin level, PESI score, and dyspnea in APE risk stratification through multivariate analysis. These findings were consistent with the conclusions of previous studies. RV/LV, as a key imaging marker for assessing right ventricular dysfunction, has been identified in multiple studies^[20–22]^ as an independent predictor of mortality in patients with APE. Furlan et al^[23]^ reported that an RV/LV > 1 ratio demonstrated sensitivity and specificity exceeding 85% for diagnosing APE complicated by right heart failure. The pathophysiological basis linking RV elevation to a poor prognosis lies in the dysregulation of right ventricular compensatory mechanisms caused by APE pressure elevation. Elevated troponin levels, a sensitive biomarker of myocardial microdamage, are associated with short-term mortality, related deaths, and adverse outcomes in patients with APE.^[24]^ Fu et al^[25]^ similarly found that cardiac troponin levels were significantly elevated in patients with APE and were involved in disease progression and risk stratification. The PESI integrates multiple clinical parameters to establish a systematic risk assessment framework that demonstrates good clinical utility and discriminatory performance.^[26]^ A previous study^[27]^ confirmed that the PESI scoring system not only accurately assessed the severity of APE, but also effectively evaluated in-hospital mortality and 30-day mortality, with both sensitivity and specificity exceeding 90%. Chan et al^[28]^ similarly concluded that higher PESI scores indicate more severe disease, shorter survival times, and poorer prognosis. This study constructed a clinical nomogram prediction model integrating the PESI scores, troponin levels, and dyspnea symptoms. The model demonstrated excellent and stable discriminatory performance, with AUC values of 0.834 and 0.805 in the training and validation cohorts, respectively.

The innovation of this study lies in the employing the optimal modeling algorithm (LR) selected through comparative evaluation of multiple algorithms to systematically integrate thrombus radiomics features, a microscopic imaging biomarker, with the aforementioned macroscopic and functional indicators. The results showed that the AUC value of the combined model was significantly higher than that of the single model. Furthermore, in both the training and validation cohorts, the ROC curves exhibited statistically significant differences compared to each individual model (*P* < .05). The DCA indicated that the combined model yielded the highest net clinical benefit when the risk threshold exceeds 0.04. This discovery has important guiding significance in practice and is expected to provide clinicians with quantitative decision support and help formulate individualized treatment strategies, thereby optimizing the allocation of medical resources and improving patient prognosis. Furthermore, the calibration curve demonstrated excellent consistency between the predicted probabilities of the combined model and actual observed outcomes, further enhancing its credibility and applicability in clinical practice. This study not only achieved significant improvements in statistical performance, but also represents a major breakthrough in the concept of risk assessment. Traditional risk assessment models primarily focus on the “downstream effects” of thrombus formation (such as right ventricular dysfunction), whereas our combined model incorporated “upstream factors, ”namely the heterogeneity of the thrombus itself. This study provides a novel imaging approach to deepen our understanding of the pathophysiological mechanisms underlying APE.

This study had certain limitations. This was a single-center retrospective study, and the sample size may have been insufficient to fully support effective learning of all features. As a result, the model may be prone to overfitting the training data, which could reduce generalization ability and affect the stability and reliability of the study’s conclusions. In future studies, redundant features could be appropriately removed and the feature dimensions could be reduced. Concurrently, multicenter prospective studies with large sample sizes should be conducted for external validation, thereby further enhancing the stability, generalizability, and clinical utility of the findings. This study employed a binary stratification model that essentially represented a simplified version of the European Society of Cardiology consensus classification. Although this strategy effectively optimized the balance of sample sizes between the each group and clinical utility with statistical power, it obscured the risk differences between the low-to-moderate- and moderate-to-high-risk groups, failing to adequately distinguish the risk characteristics and intervention needs of different subgroups. Therefore, future studies should expand the sample size, with a particular focus on increasing the proportion of high-risk patients, avoiding merging subgroups, constructing a more refined multilevel risk stratification system, and incorporating additional indicators capable of distinguishing risk differences among subgroups. Multi-classification prediction can more accurately capture the characteristic differences among various risk subgroups, clearly distinguish the management needs of patients in each group, and provide a reliable basis for developing personalized intervention strategies in clinical practice. Radiomics features extraction relies on manual ROI delineation of 2-dimensional images. Although this process demonstrated good consistency via ICC validation, it remains subjective, time-consuming, and laborious, limiting its efficiency and standardization in large-scale clinical applications. Future advancements in automated and standardized image segmentation techniques could reduce errors and enhance feature consistency and reproducibility.^[29]^

## 5. Conclusion

This study demonstrated that the predictive model developed using LR algorithm achieved optimal performance, and successfully established and validated a combined model integrating clinical characteristics, conventional CTPA imaging parameter, and thrombotic radiomics features, which demonstrated outstanding performance in predicting risk stratification in patients with APE. This combined model holds promise as a reliable adjunct tool for APE risk stratification and aids clinicians in formulating individualized treatment plans. Future multicenter prospective studies are required to validate the generalizability and clinical utility of our findings.

## Author contributions

**Data curation:** Min Wang, Yang Lu.

**Investigation:** Yaxi Yu.

**Methodology:** Fei Yang.

**Software:** Rong Chen.

**Supervision:** Dawei Wang.

**Writing – original draft:** Jianxia Song.

**Writing – review & editing:** Fei Wang.
